# Stachyose Improves the Effects of Berberine on Glucose Metabolism by Regulating Intestinal Microbiota and Short-Chain Fatty Acids in Spontaneous Type 2 Diabetic KKAy Mice

**DOI:** 10.3389/fphar.2020.578943

**Published:** 2020-10-22

**Authors:** Hui Cao, Caina Li, Lei Lei, Xing Wang, Shuainan Liu, Quan Liu, Yi Huan, Sujuan Sun, Zhufang Shen

**Affiliations:** State Key Laboratory of Bioactive Substance and Function of Natural Medicines, Institute of Materia Medica, Chinese Academy of Medical Sciences & Peking Union Medical College, Beijing, China

**Keywords:** stachyose, berberine, gut microbiota, short-chain fatty acids, glucose metabolism, type 2 diabetes

## Abstract

Berberine (BBR) has the beneficial effects of anti-inflammation, anti-bacteria, and anti-diabetes. The clinical application of BBR has been hindered by its poor gastrointestinal absorption. Stachyose (Sta), a prebiotic agent, improves the composition of gut microbiota and benefits for diabetes. We therefore investigated whether Sta improves the anti-diabetic actions of BBR using KKAy mice. Here, we find that the combination of BBR and Sta is more effective than BBR alone in blood glucose control, improvement of insulin resistance and islet functions, inflammatory mediators decrease, and maintenance of intestinal barrier integrity. Gut microbiota analysis demonstrates that both BBR and combined administration enhance the abundance of Bacteroidaceae and Akkermansiaceae and decrease Lachnospiraceae levels, whereas Akkermansiaceae elevation due to the administration of BBR with Sta is more significant than BBR alone. Interestingly, the proportion of Lactobacillaceae increases with combination treatment, but is diminished by BBR. Additionally, BBR with Sta significantly reduces the concentrations of fecal short-chain fatty acids compared to BBR. Collectively, these results indicate that the combination of BBR and Sta imparts better effects on the maintenance of glycemia and intestinal homeostasis than BBR alone by modulating gut microbiota and short-chain fatty acids, thereby providing a novel approach for the treatment of type 2 diabetes mellitus.

## Introduction

Type 2 diabetes mellitus (T2DM) is a metabolic disease characterized by obesity, insulin resistance, chronic hyperglycemia, low-grade inflammation, alteration of gut microbiota, and islet β cell dysfunction (Schwarz et al., 2013). T2DM, which accounts for 90% of diabetes cases, is a major economic burden and life pressure on people. In addition, the incidence of diabetes continues to increase each year. Hence, it is essential to identify new preventive approaches to inhibit the progression of T2DM and control the growing epidemic.

Traditional Chinese medicine has been used in clinic for over 2,000 years. The Qianjin huanglian pill, which is composed of *Coptis chinensis* powder and fresh *Rehmanniae radix*, is a classic ancient prescription for treating diabetes due to its effects of nourishing Yin and clearing heat. Berberine (BBR), one of the main components of *R. coptidis*, is an isoquinoline alkaloid originally isolated from *C. chinensis* (Jia et al., 2017). Several studies have shown that BBR has many advantageous biological roles in preclinical and clinical research, such as anti-inflammation, anti-insulin resistance, anti-hyperglycemia, anti-hyperlipidemia, anti-bacteria, and anti-diabetes (Chang et al., 2015b; Li et al., 2019; Neag et al., 2018). BBR also ameliorates liver, cardiovascular, and renal disease that are associated with diabetes (Chang et al., 2015b; Neag et al., 2018). This evidence suggests that BBR is a potential drug for the treatment of T2DM (Belwal et al., 2020). However, BBR may result in gastrointestinal reactions (including diarrhea and constipation) in some patients due to its poor absorption, thereby limiting its long-term and wide application in T2DM management (Chang et al., 2015b; Imenshahidi and Hosseinzadeh, 2019).

A plethora of studies has revealed that prebiotics not only reduce the inflammatory response and oxidative stress mainly by enhancing the growth of specific beneficial bacteria found in the gut, but also improve intestinal tight junction integrity and decrease intestinal permeability by increasing the expression of adhesion proteins within the intestinal epithelium, consequently preventing the occurrence and development of T2DM (Nakamura and Omaye, 2012; Gomes et al., 2014). Furthermore, prebiotics have the ability to promote the absorption of antioxidants (Gomes et al., 2014). Reports have also shown that BBR exhibits anti-diabetic actions by regulating gut microbiota composition and short-chain fatty acids (SCFAs) (Han et al., 2011; Liu et al., 2018). On the basis of these findings, we hypothesized that usage of a prebiotic during BBR treatment may optimize glucose metabolism and better protect against diabetes.

Stachyose (Sta) is a functional oligosaccharide extracted from fresh *R. radix*. As a classic prebiotic, Sta has been proven to promote the growth of beneficial intestinal bacteria, inhibit pathogenic bacteria, and improve glucose metabolism (Liu et al., 2019; Liang et al., 2020). Moreover, Sta enhances the absorption of soybean genistein and tea polyphenols in mice (Li et al., 2016; Lu et al., 2017). Therefore, we hypothesized that Sta improves the actions of BBR on glucose homeostasis by regulating intestinal microbiota. This study aimed to investigate the effects and mechanisms of BBR combined with Sta on glucose metabolism, inflammation modulation, intestinal integrity, fecal SCFAs, and gut microbiota in diabetic KKAy mice.

## Materials and Methods

### Chemical Compounds

Sta (purity > 80%) was provided by the laboratory of Professor Dequan Yu, Institute of Materia Medica, Chinese Academy of Medical Sciences, Peking Union Medical College (Beijing, China). BBR (purity > 98%) was obtained from the Northeast General Pharmaceutical Factory (Shenyang, China).

### Animals and Experimental Design

Eight-week-old female KKAy mice were purchased from Beijing Huafukang Bio-Technology Co., Ltd., and maintained at Experimental Animal Center of the Institute of Materia Medica under specific pathogen-free conditions. Animals were kept at 23 ± 2°C in a 12-h light/dark cycle with free access to food and water. Mice were fed with high-fat diets (45% of energy from fat; D12451; Research Diets, USA). Animal experiments were performed in accordance with the “3R” principles and guidelines for laboratory animals (GB14925-2001 and MOST 2006a) established by the People’s Republic of China, and approved by the Institutional Animal Care and Use Committee of Institute of Materia Medica (Chinese Academy of Medical Sciences and Peking Union Medical College, Beijing, China).

After 6 weeks of high-fat diets feeding, KKAy mice were randomly divided into four groups (*n* = 12) according to the levels of blood glucose, triglyceride, total cholesterol, body weight, and percentage of glycemia reduction at 40 min after insulin injection: control group (Con), Sta-treated group (Sta, 200 mg/kg), BBR-treated group (BBR, 100 mg/kg), and BBR with Sta-treated group (BBR + Sta; Sta: 200 mg/kg, BBR: 100 mg/kg). All mice were treated intragastrically with compounds or an equivalent volume of water once daily for 8 weeks.

### Fasting Blood Glucose and Glycated Hemoglobin Measurements

Fasting blood glucose (FBG) levels were measured using the glucose-oxidase method (Biosino Bio-Technology and Science Inc., Beijing, China). After 35 days of treatment, glycated hemoglobin (HbA1c) levels were assessed using commercial kits (A5911; Homa Biological, Beijing, China)

### Oral Glucose Tolerance Test, Insulin Tolerance Test, and Glucose-Stimulated Insulin Secretion Test

For oral glucose tolerance test (OGTT), mice were fasted for 4 h and given d-glucose (2 g/kg) intragastrically, and blood glucose was measured at 0, 15, 30, 60, and 120 min after glucose administration. For insulin tolerance test (ITT), mice were injected subcutaneously with insulin (0.4 U/kg) after 4 h of fasting, and blood glucose was measured at 0, 40, and 90 min after insulin injection. For glucose-stimulated insulin secretion test, mice were treated with d-glucose (2 g/kg) after 4 h of fasting, and insulin levels in plasma were monitored at 0 and 15 min after glucose administration.

### Immunofluorescence Assay

All mice were sacrificed through cervical dislocation and the pancreas were fixed in 10% formalin, embedded in paraffin, and dissected to prepare 5-μm-thick slides, then stained with antibodies against insulin (MAB1417; R&D Systems, USA) and glucagon (ab92517; Abcam, USA) as previously described (Li et al., 2017). The mean fluorescence intensities of insulin and glucagon, which account for islets areas were quantified using Image J software.

### Enzyme-Linked Immunosorbent Assay

The levels of interleukin (IL)-10 (M1000B), IL-1β (MHSLB00), and IL-6 (M6000B), monocyte chemotactic protein (MCP-1; MJE00B), tumor necrosis factor (TNF)-a (MTA00B), and C-reactive protein (CRP; DY1829) in plasma were monitored using ELISA kits (R&D Systems, USA). The concentrations of glucagon (DGCG0; R&D Systems, USA) and insulin (80-INSMSU-E10; ALPCO, USA) were determined by ELISA kits according to the manufacturer’s instructions.

### Western Blotting

Intestinal tissues were homogenized and lyzed in radio-immunoprecipitation assay buffer, and protein concentrations were determined using a bicinchoninic acid protein quantitation kit. Lysate samples were fractionated by sodium dodecyl sulfate-polyacrylamide gels and transferred onto polyvinylidene difluoride membranes, blocked with 5% non-fat milk for 1.5 h at room temperature, and incubated with primary antibodies overnight at 4°C. Appropriate horseradish peroxidase (HRP)-conjugated secondary antibodies were applied for 1–2 h at room temperature, prior to detection with an enhanced chemiluminescence kit. Antibodies to anti p-p38MAPK (Thr180/Tyr182; 4511), anti-*p*-ERK1/2 (Thr202/Tyr204; 9101), anti-*p*-JNK (Thr183/Tyr185; 4668), anti-p38MAPK (9212), anti-ERK1/2 (4695), anti-JNK (9252), anti-TLR4 (14358), anti-CD11c (97585), and anti-F4/80 (30325) were obtained from Cell Signaling Technology (CST, Danfoss, MA, USA). Antibodies against zonula occludens-1 (ZO-1, 61-7300) and occludin (33-1500) were purchased from Invitrogen (USA). β-Actin antibody (C1313), goat anti-rabbit IgG/HRP (C2226), and goat anti-mouse IgG/HRP (C2225) were purchased from Applygen Technologies Inc. (Beijing, China). Protein levels were normalized to those of β-actin.

### Quantitative Real-Time PCR

Quantitative real-time PCR was conducted as previously described (Chang et al., 2015a). Briefly, RNA was isolated from intestinal tissues using TRizol reagent (15596018; Life Technology, USA) and reverse transcribed with TransScript® first-strand cDNA Synthesis SuperMix (AT311; TransGen Biotec, Beijing, China) based on the manufacturer’s protocols. Quantitative real-time PCR was conducted using TransStart® Tip Green qPCR SuperMix (AQ141; TransGen Biotec, Beijing, China) on 7900 Real-Time PCR System (Applied Biosystems, USA). Gene expression levels were normalized to those of β-actin. The primer sequences used in this study are shown in Supplementary Table S1.

### Flow Cytometry Analysis

Cell suspensions were prepared from mesenteric lymph nodes of KKAy mice according to previous report (Moro et al., 2015). Single-cell suspensions were stained with antibodies against the following cell surface antigens: fluorescein isothiocyanate (FITC)-conjugated anti-CD11b (557396; BD, USA), phycoerythrin (PE)-conjugated anti-MHC-II (107608; Biolegend, USA), PE-CY7-conjugated anti-CD11c (558097; BD, USA), PE-CY5.5-conjugated anti-CD45.2 (109828; Biolegend, USA), and allophycocyanin (APC)-conjugated anti-F4/80 (123116; Biolegend, USA). Samples were detected on a NovoCyte flow cytometer (ACEA Biosciences, China), and the data were analyzed with NovoExpress flow cytometry analysis software (ACEA Biosciences, China).

### Gut Microbiota Profiling

The intestinal flora in feces was assayed as previously described (Jiang et al., 2018). Fecal samples were collected and snap-frozen in liquid nitrogen, followed by storage at −80°C. Genomic DNA was isolated with DNA isolation kit. The V3–V4 region of 16S rRNA genes were amplified and purified. Subsequently, the abundance and diversity of gut microbiota were analyzed using Illumina MiSeq sequencing (Major Bio-Pharm Technology, Shanghai, China) according to the standard protocol. The effective reads from all samples were grouped into operational taxonomic units (OTUs) on the basis of 97% sequence similarity. α-Diversity was estimated by the level of OTUs, and Shannon and Chao indices. β-Diversity was assessed by computing for unweighted UniFrac and visualized by principal coordinate analysis (PCoA). The sequence data were processed and analyzed on the free online Majorbio I-Sanger Cloud Platform (www.i-sanger.com).

### Short-Chain Fatty Acids Analysis

SCFAs in fecal samples were detected on the basis of previous report (Liao et al., 2019). Briefly, frozen feces were crushed with methanol containing internal standards, subjected to ultrasonication for 30 min, centrifuged for 15 min, and extracted. Subsequently, the SCFAs in each sample were assayed by gas chromatography coupled to a mass spectrometer detector (GC-MS) (Agilent Technologies Inc. CA, USA) and quantified using Masshunter quantitative software.

Correlation analysis of SCFAs and gut microbiota were performed on the platform of Majorbio I-Sanger Cloud (www.i-sanger.com). R and *p* values were obtained using Spearman’s rank correlation.

### Statistical Analysis

The data are presented as the mean ± SEM. Statistical analysis was performed using GraphPad Prism 7.0. Differences in FBG, OGTT, ITT, body weight, food intake, and water intake were assessed using two-way analysis of variance (ANOVA) with Tukey’s test. Data sets involved in two groups or multiple groups were analyzed using unpaired two tailed Student’s *t*-test or one-way ANOVA depending on the experiments. Differences with *p* < 0.05 were considered statistically significant.

## Results

### Effects of Berberine With Stachyose on Glucose Homeostasis

**FIGURE 1:**
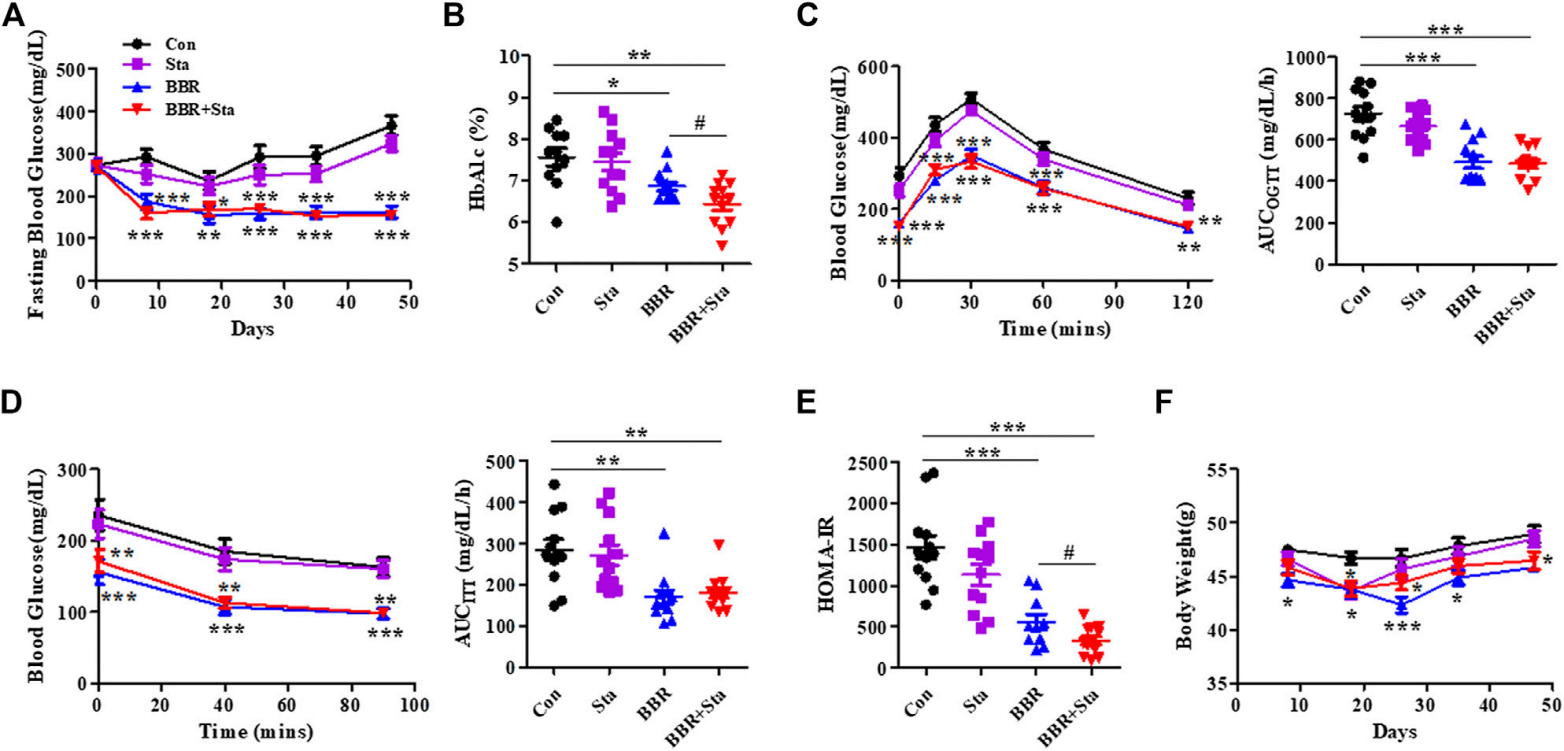
BBR combined with Sta improves glucose metabolism in KKAy mice. **(A)** Fasting blood glucose. **(B)** HbA1c levels. **(C)** Oral glucose tolerance test (OGTT). **(D)** Insulin tolerance test (ITT). Area under the curve (AUC) of OGTT or ITT. **(E)** Homeostasis model assessment of insulin resistance (HOMA-IR) index. The index of the validated HOMA-IR was calculated as follows: HOMA-IR = fasting blood glucose (mmol/L) × fasting plasma insulin (μU/mL)/22.5. **(F)** Body weight of mice. Data are expressed as mean ± SEM, *n* = 10–12. *p < 0.05, **p < 0.01, ***p < 0.001 vs. Con; ^#^ *p* < 0.05 vs. BBR. BBR, berberine; Con, control; Sta, stachyose.

In this study, we found that the levels of blood glucose and HbA1c in mice treated with either BBR or BBR+Sta were significantly decreased compared to control mice (Figures 1A,B). Further, HbA1c levels in combination-treated mice obviously lowered compared to those of BBR-treated mice (Figure 1B), indicating that BBR combined with Sta is more effective in glycemic control than BBR alone.

As depicted in Figures 1C,D, in comparison to the Con mice, supplementation with BBR or BBR with Sta improved glucose tolerance and insulin sensitivity, as evaluated by diminished AUCs for blood glucose during OGTT and ITT. We further detected the index of HOMA-IR to estimate insulin resistance levels. The HOMA-IR indices in Sta, BBR, and BBR with Sta-treated mice were decreased by 21.9% (*p* = 0.0880), 61.2% (*p* < 0.001), and 79.3% (*p* < 0.001), respectively, compared to the Con mice (Figure 1E), which implied that BBR with Sta is better in improving insulin sensitivity of KKAy mice than BBR alone.

In addition, treatment with BBR or BBR with Sta resulted in lowered weight gain, food consumption, and water intake (Figure 1F; Supplementary Figure S1). Therefore, the effects of BBR and BBR with Sta on body weight may be in correlation with decreased food intake. We noticed that the food and water intake of mice had no distinction between BBR and combination groups, but the weight of mice administrated of BBR with Sta had an increased tendency in comparison to that of BBR treatment at eighth, 26th, and 35th days, indicating that Sta may attenuate the gastrointestinal side effects of BBR and bring about mild weight increase.

### Effects of Berberine With Stachyose on Islet Functions

**FIGURE 2:**
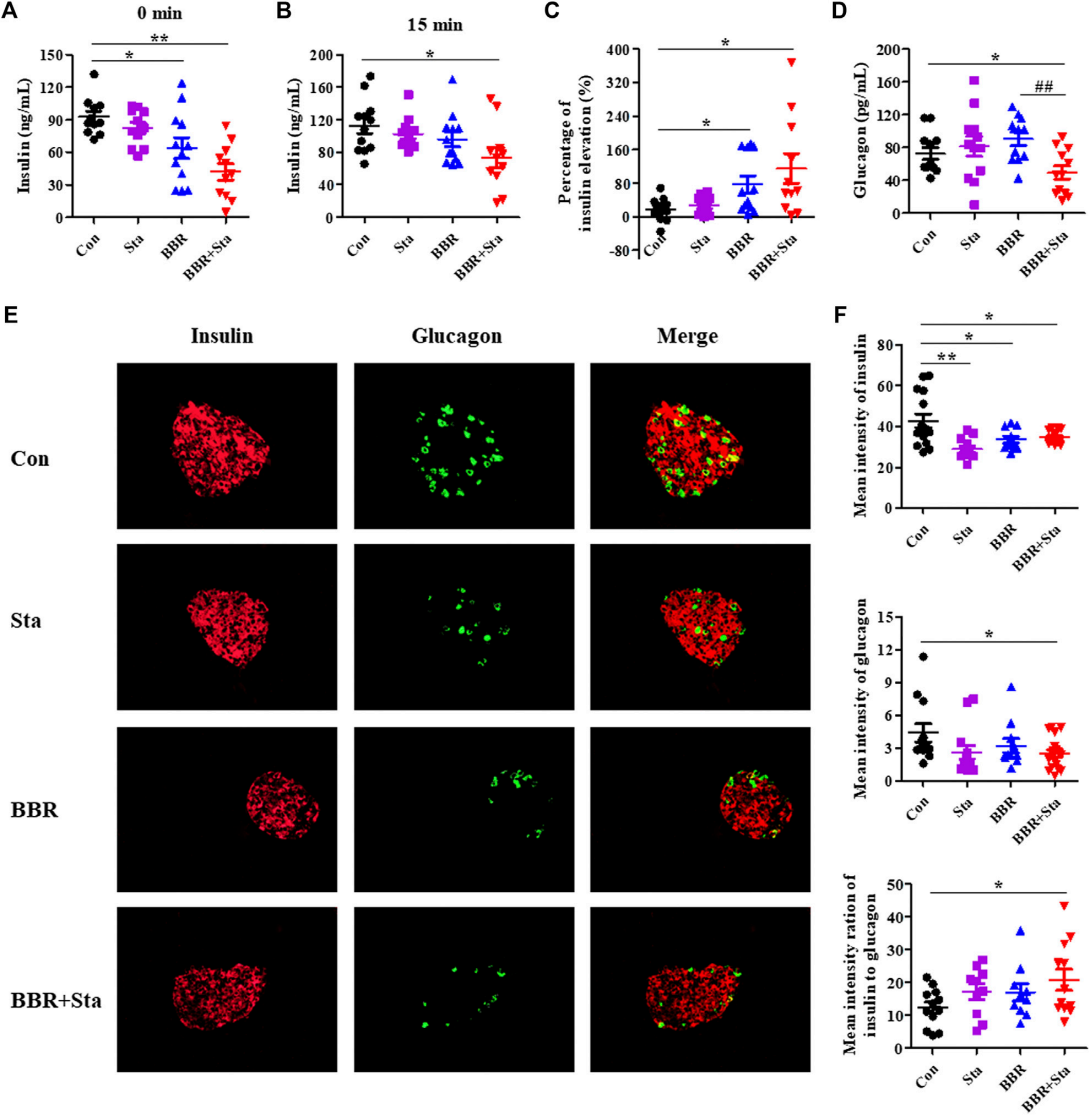
BBR combined with Sta modifies islet functions in KKAy mice. **(A)** Fasting insulin levels in plasma. **(B)** Plasma insulin levels after glucose administration for 15 min **(C)** The percentage of insulin elevation after glucose administration for 15 min **(D)** Plasma glucagon concentrations. **(E)** Representative images of insulin and glucagon immunofluorescence staining in pancreatic islets. Insulin is shown in red, and glucagon in green. Magnification of all images is ×400. **(F)** Statistical analysis of the mean fluorescence intensities of insulin and glucagon in islets, and the ratio of insulin to glucagon. Data are presented as mean ± SEM, *n* = 10–12 for **(A–D)**; *n* = four to six per group for **(E, F)**. *p < 0.05, **p < 0.01, ***p < 0.001 vs. Con; ^#^ *p* < 0.05, ^##^ *p* < 0.01 vs. BBR. BBR, berberine; Con, control; Sta, stachyose.

As shown in Figure 2A, compared to the Con group, fasting plasma insulin levels in the BBR and BBR with Sta groups were reduced by 27.8% (*p* < 0.05) and 45.7% (*p* < 0.01), respectively. Besides, BBR with Sta treatment promoted insulin release and increased proportion of insulin elevation from 17.5% in Con mice to 118.2% (*p* < 0.05), while BBR treatment elevated to 76.0% (*p* < 0.05) (Figures 2A–C). And the concentration of plasma glucagon was only reduced by the combined treatment in comparison to Con or BBR supplementation (Figure 2D).

Compared to the Con group, glucagon contents in islets were decreased in the BBR combined with Sta group and had no obvious alteration in the BBR group, and insulin levels in the islets of all treatment groups were declined (Figures 2E,F). Additionally, the mean fluorescence intensity ratio of insulin to glucagon was markedly increased in the combination group (Figure 2F). Taken together, these results suggest that BBR combined with Sta is more effective than BBR alone in maintaining insulin-glucagon homeostasis and ameliorating islets functions.

### Effects of Berberine With Stachyose on Inflammation

**FIGURE 3:**
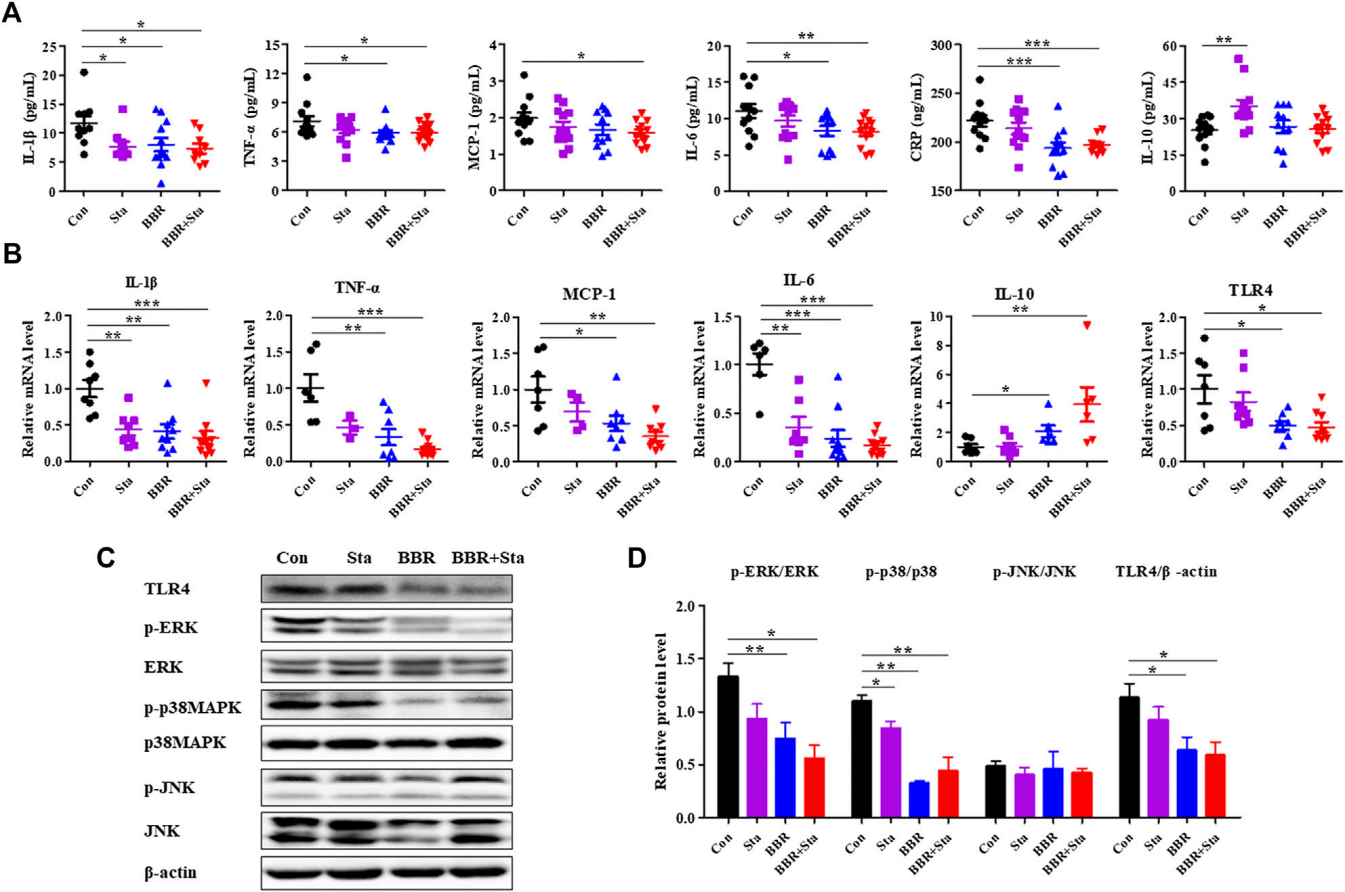
BBR combined with Sta improves inflammatory status in KKAy mice. **(A)** Cytokine levels of interleukin (IL)-1β, tumor necrosis factor (TNF)-α, monocyte chemotactic protein (MCP)-1, IL-6, C-reactive protein (CRP), and IL-10 in plasma were detected by ELISA. **(B)** Gene expression levels of IL-1β, TNF-α, MCP-1, IL-6, IL-10, and TLR4 in intestine were analyzed by qPCR. **(C, D)** Protein levels of TLR4, p38MAPK, p-p38MAPK, ERK1/2, *p*-ERK1/2, JNK, and *p*-JNK were determined by Western blot analysis. β-Actin was detected as the internal reference. Data are expressed as mean ± SEM, *n* = 10–12 for **(A)**; *n* = four to five for **(B–D)**. *p < 0.05, **p < 0.01, ***p < 0.001 vs. Con. BBR, berberine; Con, control; Sta, stachyose.

Compared with the Con mice, treatment with BBR and BBR with Sta diminished cytokine levels of IL-1β, TNF-a, IL-6, CRP in plasma, Sta reduced IL-1β levels and increased IL-10 levels, while the production of MCP-1 was only decreased by BBR with Sta treatment (Figure 3A).Similarly, the gene levels of pro-inflammatory cytokines- IL-1β, TNF-a, MCP-1, and IL-6-were attenuated by BBR and BBR with Sta, whereas anti-inflammatory cytokine IL-10 levels in intestine was elevated (Figure 3B). And the actions induced by the combination treatment were more pronounced (Figure 3B). Given that the production of proinflammatory cytokines is related to the activation of the TLR4 and MAPK signaling pathways (Lontchi-Yimagou et al., 2013), we next examined whether these pathways in intestinal tissues were impacted by BBR with Sta treatment. As shown in Figures 3B–D, compared to the Con group, the protein and mRNA levels of TLR4 as well as the phosphorylation of ERK and p38MAPK were all suppressed in the BBR and BBR with Sta groups, without obvious change of JNK phosphorylation.

### Effects of Berberine With Stachyose on the Proportion and Function of Macrophage

**FIGURE 4:**
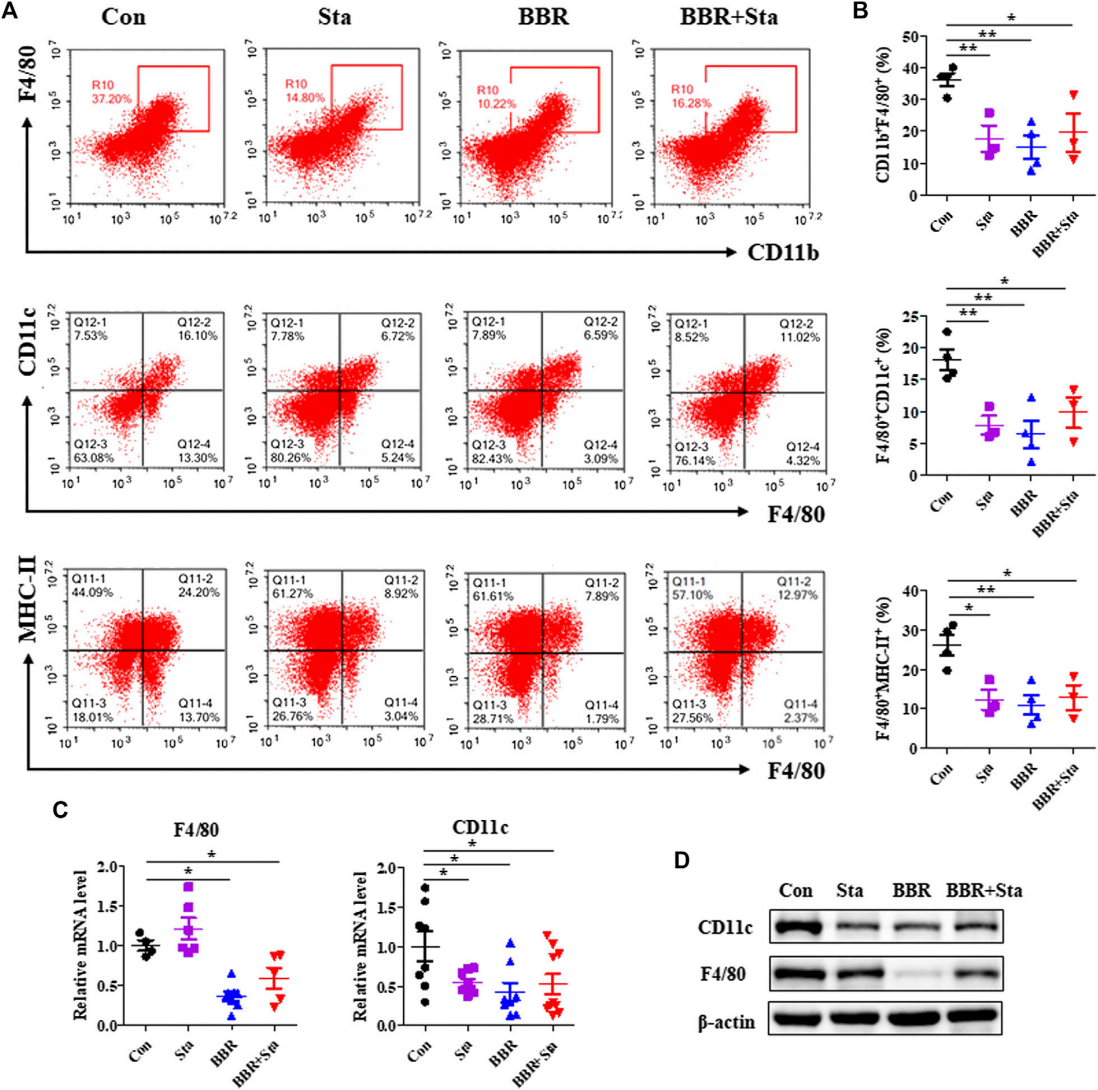
Effects of BBR with Sta on the proportion and function of macrophage. **(A)** Representative FACS staining images for CD11b, CD11c, F4/80, and MHC-II on gated CD45.2^+^ cells in mesentery. **(B)** Statistical analysis of the percentage of macrophage (CD11b^+^F4/80^+^), M1 macrophage (F4/80^+^CD11c^+^), and MHC-II expressed on macrophage surface (F4/80^+^MHC-II^+^). **(C)** Gene expression levels of F4/80 and CD11c in intestine tissues were measured by quantitative real-time PCR. **(D)** Protein levels of CD11c and F4/80 in intestine were detected by Western blot. β-Actin as the internal reference. Data are presented as mean ± SEM, *n* = 3–5. *p < 0.05, **p < 0.01 vs. Con. BBR, berberine; Con, control; Sta, stachyose.

A number of studies have demonstrated that macrophages participate in the occurrence and development of T2DM (Frydrych et al., 2018), and thus we investigated whether macrophages are related to the properties of hypoglycemia and anti-inflammation exerted by BBR with Sta. We observed that in mesenteric lymph nodes, the percentage of macrophages (CD11b^+^F4/80^+^) and M1 macrophages (F4/80^+^CD11c^+^) as well as the level of MHC-II expressed on macrophages surface were significantly decreased in all treatment groups compared with the Con group (Figures 4A,B). Similarly, the protein and mRNA levels of F4/80 and CD11c in intestines were also reduced by BBR and BBR with Sta treatments, and Sta lowered CD11c gene level and protein expressions of F4/80 and CD11c (Figures 4C,D).

### Effects of Berberine With Stachyose on Intestinal Integrity

**FIGURE 5:**
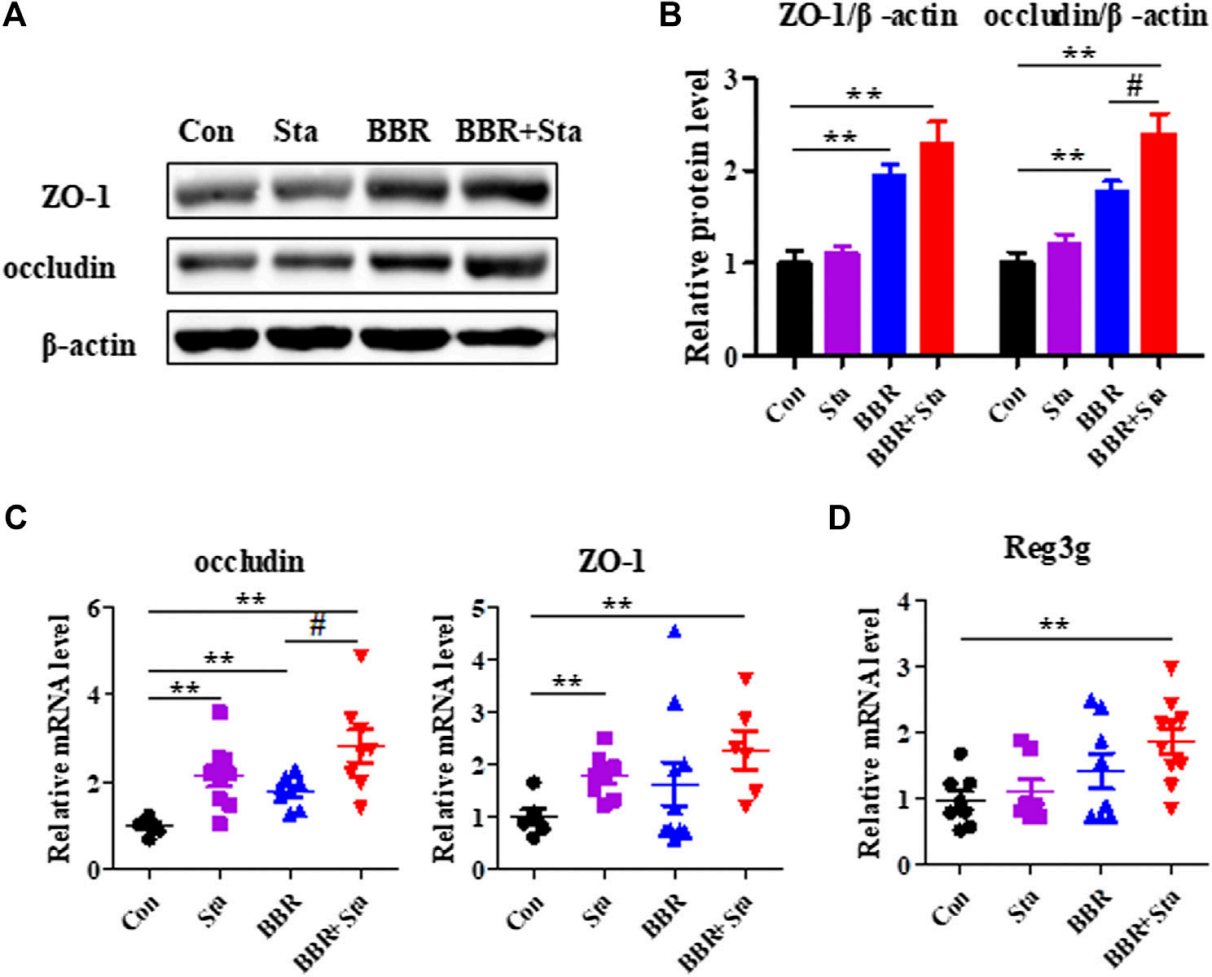
BBR with Sta maintains intestinal barrier integrity of KKAy mice. **(A)** Protein levels of ZO-1 and occludin in intestine tissues were analyzed by Western blot. **(B)** Quantization of ZO-1 and occludin proteins in intestine. **(C, D)** Gene expression levels of occludin, ZO-1, and Reg3g in intestine were evaluated by quantitative real-time PCR. β-Actin was detected as the internal reference. Data are represented as mean ± SEM, *n* = 4–5. *p < 0.05, **p < 0.01 vs. Con; ^#^ *p* < 0.05 vs. BBR. BBR, berberine; Con, control; Sta, stachyose.

Given that inflammation contributes to intestinal dysbiosis and damages gut permeability, we explored the intestinal integrity of KKAy mice. Compared with the Con group, the protein and gene expression levels of ZO-1 and occludin, which are tight junction components, were upregulated in the BBR and BBR with Sta groups, and Sta remarkably elevated ZO-1 and occludin gene levels (Figures 5A–C). Moreover, relative to the Con mice, the mRNA expression of Reg3g (regenerating islet-derived three gamma), an antimicrobial protein was increased by 1.4-fold (*p* > 0.05) and 1.7-fold (*p* < 0.01) in mice treated with BBR and BBR with Sta, respectively (Figure 5D). These data indicate that BBR combined with Sta is more effective than BBR alone in maintaining gut barrier integrity and intestinal balance.

### Effects of Berberine With Stachyose on Gut Microbiota

**FIGURE 6:**
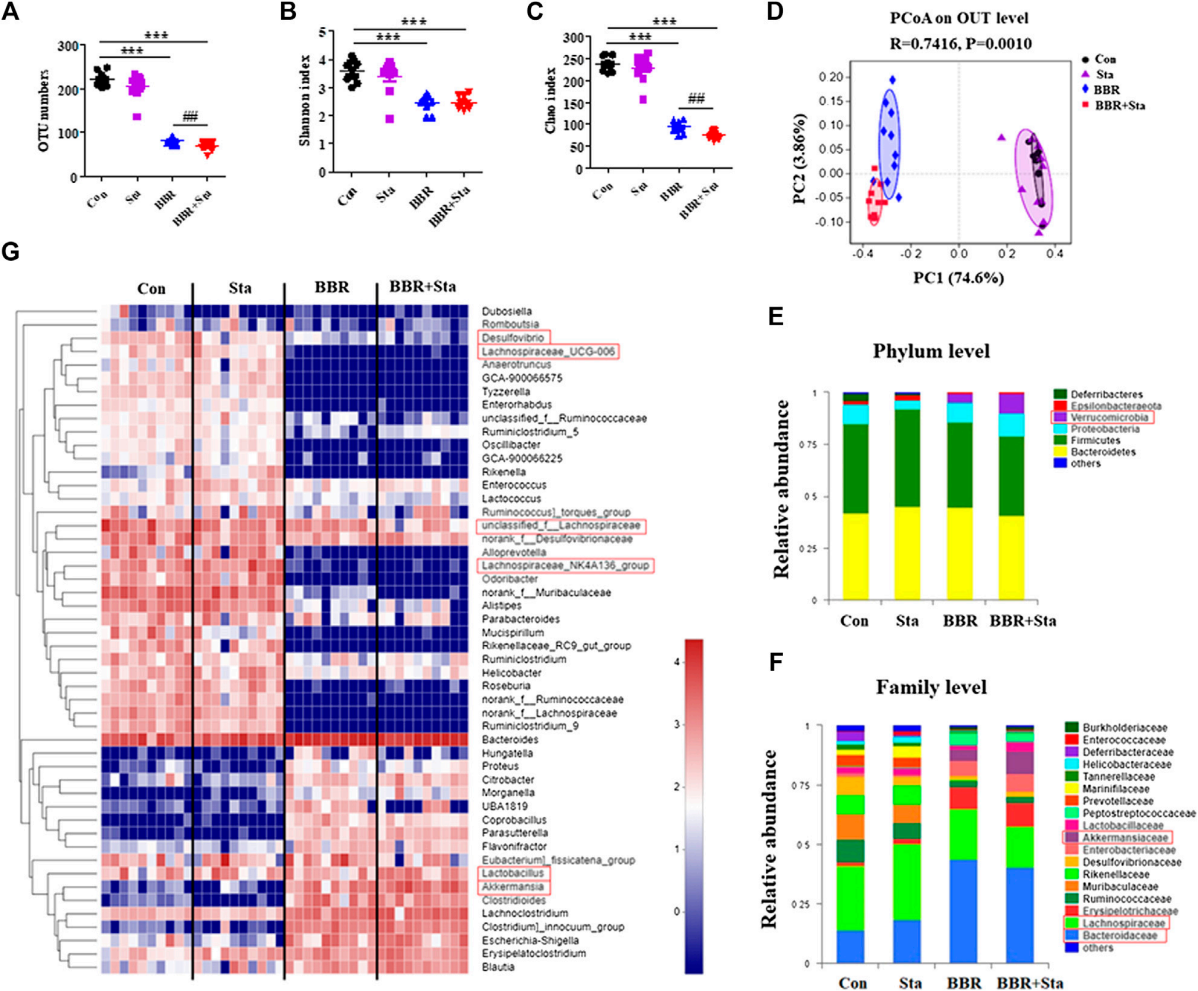
BBR combined with Sta alters the composition of gut microbiota of KKAy mice. **(A)** Total OTU numbers. **(B)** Shannon index. **(C)** Chao index. **(D)** Principal coordinate analysis (PCoA). **(E)** Relative abundance of microbiota at the phylum level. **(F)** Relative abundance of microbiota at the family level. **(G)** Heatmap analysis of relative abundance of microbiota at the genus level. The heatmap shows the top 50 genera ranked on the basis of abundance. Each column in the heatmap represents one sample, and each row represents one genus. The color bar showing blue (low) to red (high) indicates the relative abundance of each genus. Data are shown as mean ± SEM, *n* = 9–10. ***p < 0.001 vs. Con; ^##^ *p* < 0.01 vs. BBR. BBR, berberine; Con, control; Sta, stachyose.

We next examined the roles of BBR with Sta on intestinal microbiota composition by performing Illumina-sequencing based analysis of bacterial 16S rRNA in fecal samples. After removing unqualified sequences, a total of 35,299 valid sequences were generated and clustered into 316 OTUs according to the minimum sample-sequence number (Supplementary Table S2). Compared to the Con group, the OTU numbers were reduced in the BBR and BBR with Sta groups (Figure 6A). And the OTU numbers were remarkably less in the combined group than those of the BBR group (Figure 6A). The Shannon and Chao indices reflect the diversity and richness of gut microbiota, respectively. As shown in Figures 6B,C, both BBR and BBR with Sta diminished the indices of Shannon and Chao, whereas there were notable differences in the Chao index between the BBR and BBR with Sta groups (*p* < 0.01).

Unweighted Unifrac PCoA based on OTU levels revealed a distinct clustering of microbiota composition in each group (Figure 6D). Multivariate ANOVA of PCoA matrix scores revealed that the microbiota community of mice in BBR and combination groups is clearly differed from that of the Con mice. The gut microbiota of the Sta, BBR, and BBR with Sta groups could also be discriminated (Figure 6D). Additionally, the bacterial community of Sta-administrated mice was closer to that of the Con mice, which coincides with the mild actions exerted by Sta.

Taxonomic profiling at the phylum level revealed that BBR and BBR with Sta treatments elevated the abundances of Verrucomicrobia and reduced that of Deferribacteres and Epsilobacteraeota compared with the Con mice (Figure 6E). Notably, the increase in abundance of Verrucomicrobia produced by BBR with Sta was more significant than BBR alone. At the same time, Sta decreased the levels of Deferribacteres and Proteobacteria (Figure 6E). At the family level, the abundances of Bacteroidaceae and Akkermansiaceae were notably enhanced, and Lachnospiraceae and Desulfovibrionaceae were decreased in the BBR and BBR with Sta groups compared to the Con group (Figure 6F). In addition, the proportion of Akkermansiaceae in combination-treated mice was higher than that of BBR-treated mice. Strangely, the proportion of Lactobacillaceae was diminished by BBR but increased by the combination of BBR and Sta (Figure 6F). The levels of Desulfovibrionaceae were also diminished by Sta treatment. Similar results were also observed at the genus level. Combination treatment declined Desulfovibrio abundance, and increased the abundance of *Lactobacillus* and Akkermansia compared with BBR treatment (Figure 6G). Collectively, these findings implied that BBR with Sta improves the composition of gut microbiota.

### Effects of Berberine With Stachyose on Fecal Short-Chain Fatty Acids

**FIGURE 7:**
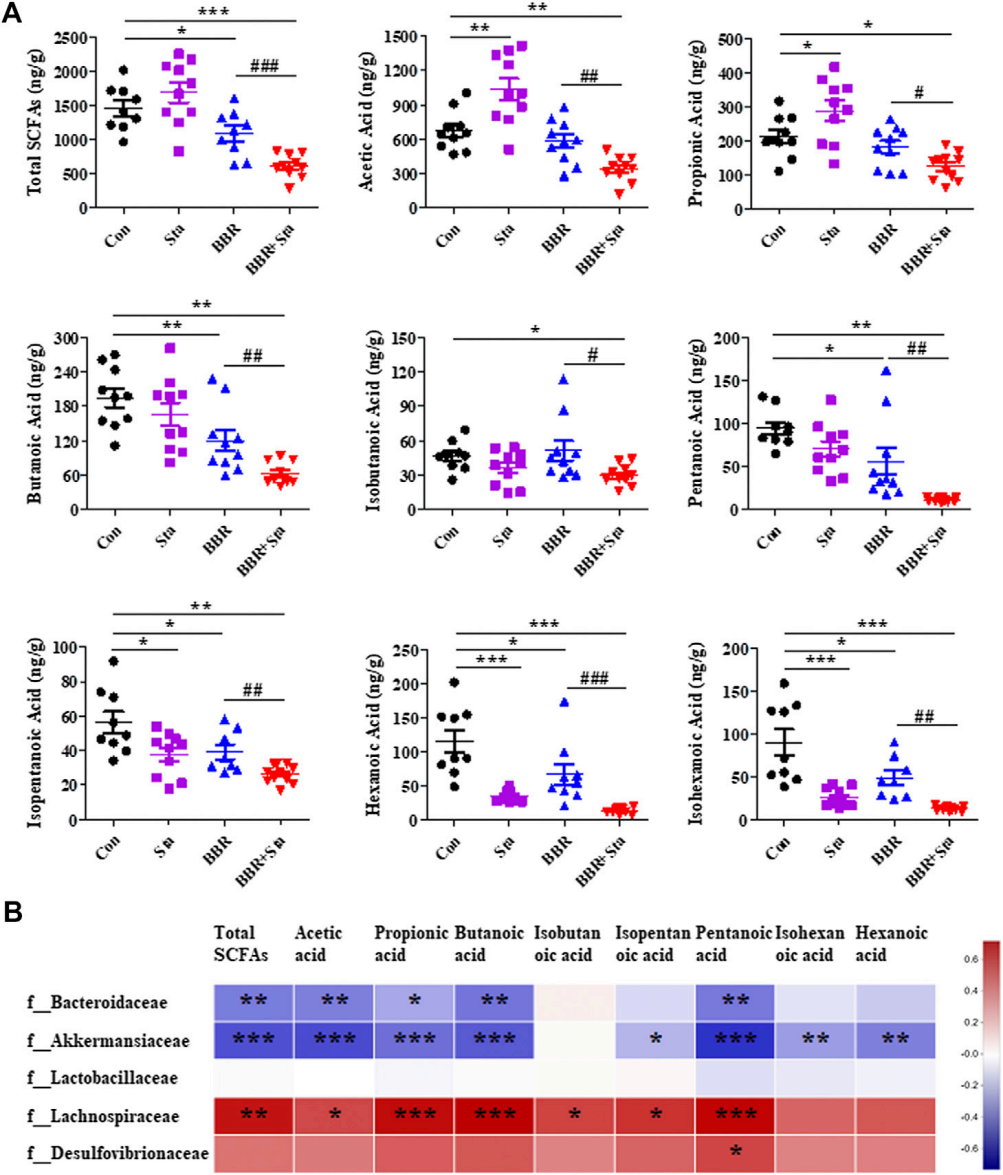
Effects of BBR with Sta on fecal short-chain fatty acids (SCFAs) of KKAy mice. **(A)** Fecal SCFAs, including acetic acid, propionic acid, butyric acid, isobutyric acid, pentanoic acid, isopentanoic acid, hexanoic acid, isohexanoic acid, were analyzed by GC-MS method. **(B)** Correlation analysis of SCFAs and specific microbiota at the family level. The R values were shown in different colors in the diagram. The blue represents negative correlation, and red represents positive correlation. *n* = 8–10 per group. For **(A)**, *p < 0.05, **p < 0.01, ***p < 0.001 vs. Con; ^#^ *p* < 0.05, ^##^ *p* < 0.01, ^###^ *p* < 0.001 vs. BBR. For **(B)**, *p < 0.05, **p < 0.01, ***p < 0.001. BBR, berberine; Con, control; Sta, stachyose.

SCFAs, including acetic acid, propionic acid, butyric acid, isobutyric acid, pentanoic acid, isopentanoic acid, hexanoic acid, isohexanoic acid, and total SCFAs, were assessed in this study. We here found that acetic and propionic acids were elevated, whereas hexanoic, isohexanoic, and isopentanoic acids decreased in the Sta group compared to the Con group **(** Figure 7A **)**. Furthermore, compared to the Con group, BBR reduced the contents of total SCFAs, butyric, pentanoic, isopentanoic, hexanoic, and isohexanoic acids in feces **(** Figure 7A **)**. In addition, the eight SCFAs and total SCFAs were all obviously downregulated in the BBR with Sta group compared with the Con or BBR group **(** Figure 7A **)**. Overall, BBR combined with Sta significantly diminished fecal SCFAs concentrations.

Subsequently, we analyzed the relationship of fecal SCFAs and intestinal bacterial at the family level (Supplementary Figure S2; Supplementary Table S3). The results displayed that Bacteroidaceae and Akkermansiaceae were negatively correlated with acetic acid, propionic acid, butyric acid, isobutyric acid, pentanoic acid, and total SCFAs, while Lachnospiraceae was positively related with these SCFAs (Figure 7B; Supplementary Table S3). And a significant positive correlation between Desulfovibrionaceae and pentanoic acid was observed (Figure 6; Supplementary Table S3). These results were consistent with the above results of microbiota and SCFAs, which suggest that BBR with Sta improves glucose metabolism and gut integrity linked to the alteration of intestinal microbiota and SCFAs.

## Discussion

Diabetes has become a major public health concern because it causes various health problems and its global prevalence has increased in recent decades (Tian et al., 2019). BBR has various pharmacological properties and multispectral therapeutic applications, including diabetes, hyperlipidemia, and metabolic syndrome (Chang et al., 2015b). Nevertheless, the use of BBR is limited due to its poor absorption and gastrointestinal adverse effects. Sta, a functional prebiotic, has been shown to improve antioxidants absorption and intestinal microbiota composition, leading to the ameliorated inflammation and diabetes (Mikelsaar and Zilmer, 2009; Liu et al., 2019; Liang et al., 2020). Here, we observed that combination of BBR and Sta is more effective than BBR alone in modulating glucose metabolism, gut inflammation, and intestinal barrier integrity by altering gut microbiota and SCFAs in the stool of KKAy mice.

T2DM is accompanied with increased levels of blood glucose and HbA1c, as well as islet dysfunction. HbA1c is an index of the control of diabetes and reflects the average blood glucose levels over the past two-three months (Mandaliya and Seshadri, 2019). Glucose homeostasis is maintained by insulin and glucagon, whereas disrupted pancreatic islet functions and insulin resistance in T2D leads to glucose intolerance (Mandaliya and Seshadri, 2019). In this study, our findings showed that the combination treatment of BBR and Sta initiated better beneficial actions on glycemia control and islets functions improvement compared to BBR alone.

It is widely accepted that T2DM is typical of chronic low-grade inflammation of metabolic tissues, such as adipose, liver, intestine, and islets (Burcelin, 2016). And various inflammation-related molecules are associated with the development of T2DM, like TLR4, MAPK, and NF-κB (Lee and Lee, 2014). Clinical studies have shown that when inflammation in T2DM patients is suppressed by a high dose of aspirin or salsalate, the glycemic control of the patients improves, along with concomitant inhibition of NF-κB activity (Lee and Lee, 2014). Our findings revealed that BBR combined with Sta imparts stronger effects on abating the expressions of proinflammatory mediators in plasma as well as in the intestines than those of BBR alone. In addition, BBR with Sta treatment downregulated the activation of TLR4 signaling pathway and the phosphorylation of ERK and p38MAPK. Overall, treatment using the combination of BBR and Sta alleviates inflammation of KKAy mice at least partially through the regulation of TLR4, ERK, and p38MAPK pathways.

Various immune cells are involved in the progression of obesity-related inflammation and T2DM, including pro-inflammatory cells (macrophages, dendritic cells, CD8^+^T cells, and B cells) and anti-inflammatory cells (regulatory T cells and eosinophils) (Lee and Lee, 2014). The number and activation of macrophages are enhanced in obese and diabetic individuals (Frydrych et al., 2018). Patsouris et al. have revealed that selective depletion of the CD11c^+^ classically M1 macrophages, reduces inflammation and improves insulin resistance (Patsouris et al., 2008). Functionally, M1 macrophages are characterized by the production of inflammatory mediators, including MCP-1, TNF-α, and IL-1β, while M2 macrophages exhibit increased production of IL-10 (Frydrych et al., 2018). Herein, we observed that the proportions of macrophages and M1 type in mesentery and intestine were lowered by BBR with Sta treatment. Simultaneously, the antigen-presenting function of macrophages was also weakened. Collectively, macrophages participate in the hypoglycemic and anti-inflammatory effects initiated by BBR with Sta.

Intestinal dysbiosis results in increased intestinal permeability and susceptibility to microbial antigens, which ultimately contribute to the occurrence and development of inflammation and diabetes (Gomes et al., 2014). ZO-1 and occludin acting as tight junction proteins play protective roles in intestinal permeability (Daly and Shirazi-Beechey, 2006). T2DM is usually accompanied with increased intestinal permeability characterized by reduction in tight junction proteins (Sharma and Tripathi, 2019). And accumulation of these molecules is associated with increased protection at the intestinal barrier level. Herein, we found that treatment with the combination of BBR and Sta produced higher expressions of occludin and ZO-1 than BBR supplementation. Sta treatment also augmented these molecules expressions. What’s more, prebiotics decrease intestinal permeability and maintain gut barrier integrity by modulating gut microbiota composition, ultimately inhibiting T2DM progression (Nakamura and Omaye, 2012). Therefore, our results suggest that Sta as a prebiotic enhances the positive effects of BBR in maintaining gut barrier integrity and amelioration of body weight loss of KKAy mice, which may be attributed to specific alterations in the gut microbiota.

The major alterations in the intestinal microbiota that are linked to T2DM include a significantly lowered prevalence of Bacteroidetes and Verrucomicrobia and an enrichment of Firmicutes, Proteobacteria, and Deferribacteres (Delzenne et al., 2015). Although the ratio of *Bacteroides* to Firmicutes was not altered in the study, *Bacteroidaceae*, belonging to *Bacteroides*, was obviously increased by BBR and BBR with Sta. Importantly, combination treatment enhanced Verrucomicrobia abundance compared with BBR alone. In line with these results, in comparison to BBR, the levels of Akkermansia and Akkermansiaceae, belonging to Verrucomicrobia, were also markedly increased by BBR with Sta. Akkermansia has beneficial effects on glucose metabolism and gut permeability, which is negatively associated with T2DM and has been tested as a probiotic in preclinical trials (Gurung et al., 2020). Plovier et al. (Plovier et al., 2017) found that pasteurized Akkermansia muciniphila vs. the live bacteria has an enhanced capacity to reduce fat mass development, insulin resistance and dyslipidemia in mice. Our previous study also manifested that BBR with Sta increased the abundances of phylum Verrucomicrobia and species Akkermansia muciniphila in the feces of db/db mice (Li et al., 2020). In addition, Verrucomicrobia is highly abundant in healthy subjects (Fujio-Vejar et al., 2017). Thus, the increased proportion of the phylum Verrucomicrobia and genus Akkermansia induced by BBR with Sta contributes to the amelioration of glucose metabolism and intestinal integrity.

*Lactobacillus* genus acting as probiotic is beneficial to healthy intestinal microbiota, creates a favorable intestinal environment, and alleviates T2DM (Salgaco et al., 2019). We observed that BBR treatment reduced the levels of Lactobacillaceae family and *Lactobacillus* genus, whereas BBR combined with Sta reversed the reduction to a higher percentage than those seen in control mice. The abundances of Desulfovibrio genus were elevated in obesity and T2DM (Van Hul et al., 2018). Desulfovibrio which is considered an opportunistic pathogen produces endotoxins and have the capacity to reduce sulfate to H_2_S, thereby damaging the intestinal barrier (Van Hul et al., 2018). We here found that both BBR and combined treatments reversed these elevations, and combined treatment diminished the Desulfovibrio genus level compared with BBR alone. Furthermore, it has been demonstrated that Akkermansia and *Lactobacillus* protect against inflammation (Gurung et al., 2020). Taken together, Sta enhances the efficacy of BBR against diabetes through altering gut bacterial composition and maintaining intestinal homeostasis.

SCFAs are closely associated with obesity and diabetes, which are products of gut microbiota mediated fermentation of resistant starch or dietary fiber (Mandaliya and Seshadri, 2019). Firmicutes and Bacteroidetes are the predominant intestinal bacteria that produce SCFAs (den Besten et al., 2013). Bacteroidetes mainly produces acetate and propionate, whereas butyrate is mostly generated by Firmicutes (den Besten et al., 2013). In this study, we found that the abundance of Firmicutes and Bacteroidetes did not significantly differ among the study groups, and thus the production of SCFAs in the mouse groups is most likely the same. Interestingly, our results showed that fecal SCFA reduction in mice that received the combination treatment was remarkably less than that of mice treated with BBR alone, which is contradictory to the results of recent studies that prebiotics and BBR elevated SCFAs concentrations to alleviate obesity and diabetes (Kim et al., 2018; Zhang et al., 2019). However, there are animal and human studies suggested that an increased concentration of SCFAs in feces was associated with higher body weight and fat gain and insulin resistance, which may be due to increased SCFAs production and decreased SCFAs absorption (Sircana et al., 2018). Therefore, decreased fecal SCFAs in KKAy mice generated by BBR with Sta is most probably beneficial in protecting against the development of T2DM. SCFAs exert a number of beneficial effects to improve gut health, insulin resistance, and diabetes, as well as enhance intestinal barrier function by regulating the expression of tight junction proteins and mucous (Dalile et al., 2019). SCFAs, particularly propionate and butyrate also inhibit the maturation of monocytes and macrophages, altering their abilities to capture antigens and reducing their ability to produce pro-inflammatory cytokines such as IL-1β, IL-6, and TNF-α (Vinolo et al., 2011; Correa-Oliveira et al., 2016). Nonetheless, most SCFAs are absorbed by the host to exert beneficial properties, and fecal SCFAs represent non-absorbed SCFAs, so it is reasonable to believe that Sta enhances the ability of BBR promoting SCFAs absorption, resulting in better effects on intestinal barrier integrity, inflammation alleviation, and glucoses metabolism. Meanwhile, it is not excluded that fecal SCFAs reduction induced by BBR with Sta treatment is attributed to lessened diversity and richness of gut microbiota and the subsequent reduction of SCFAs generation. Further investigations on the production, absorption, and excretion of SCFAs due to treatment with BBR with Sta are warranted.

In contrast to most studies on the correlation between SCFAs and gut bacteria, we found that fecal SCFAs are negatively correlated with beneficial bacteria-Bacteroidaceae and Akkermansiaceae, and positively correlated with pathogenic bacteria-Lachnospiraceae. Nevertheless, there are several reports that support our results (Turnbaugh et al., 2006; Fernandes et al., 2014). These controversial results may be explained by different models of disease, sample sources of SCFAs, and environment factors. To elucidate the effects of SCFAs and the contradictory findings about the relationship of SCFAs and intestinal microbiota in diabetes, more efforts are desired.

Notably, the beneficial effects of BBR combined with Sta on T2DM coincides with the formulation thought of ancient Chinese formula-Qianjin Huanglian pill. Our results may provide more evidence on the effect and mechanism of the Qianjin Huanglian pill against diabetes, as well as further clarify the rationality and scientific basis of the formula.

In summary, combination of Sta and BBR is more effective in glycemic control, inflammation attenuation, and gut integrity maintenance by modulating gut microbiota composition and SCFAs levels, providing a novel strategy for the treatment of T2DM. The specific mechanism of the combination treatment regulating intestinal microbial composition is unclear and requires further investigation.

## Data Availability Statement

The 16S sequencing data has been deposited in NCBI Sequence Read Archive. The (SRA) accession number is SRP270302, the bioproject accession is PRJNA644271, and the data could be found at SRA RunSelector: https://www.ncbi.nlm.nih.gov/Traces/study/?acc=PRJNA644271.

## Ethics Statement

The animal study was reviewed and approved by the Institutional Animal Care and Use Committee of Institute of Materia Medica, Chinese Academy of Medical Sciences & Peking Union Medical College, Beijing, China

## Author Contributions

HC and CL designed the research, performed the research, analyzed the data, and wrote the manuscript. LL, XW, SL, QL, YH, and SS participated in experiments. SZ supervised the whole project, supplied academic and technical support, and reviewed the manuscript. All authors read and approved the final manuscript.

## Funding

The work was supported by the National Natural Science Foundation of China (No. 81773962 and 81900480), Natural Science Foundation of Beijing Municipality (No. 7204281 and 7202137), the Drug Innovation Major Project (No. 2018ZX09711001–003-009 and 2018ZX09711001–009-014), and the CAMS Initiative for Innovative Medicine (CAMS-I2M) (No. 2016-I2M-2-006 and 2017-I2M-1-010).

## Conflict of Interest

The authors declare that the research was conducted in the absence of any commercial or financial relationships that could be construed as a potential conflict of interest.
