# Supplementary material for: Stachyose Improves the Effects of Berberine on Glucose Metabolism by Regulating Intestinal Microbiota and Short-Chain Fatty Acids in Spontaneous Type 2 Diabetic KKAy Mice
Source: Front Pharmacol. 2020 Oct 22;11:578943. doi: 10.3389/fphar.2020.578943 (PMC7642818; doi:10.3389/fphar.2020.578943)
Supplement: Supplementary file 3 [file Table3_v1.DOCX]

**Stachyose Improves** **the Effects of Berberine on Glucose Metabolism by Regulating Intestinal Microbiota and SCFAs in Spontaneous Type 2 Diabetic KKAy Mice**

Hui Cao^†^, Caina Li^†,*^, Lei Lei, Xing Wang, Shuainan Liu, Quan Liu, Yi Huan, Sujuan Sun, Zhufang Shen^*^

State Key Laboratory of Bioactive Substance and Function of Natural Medicines, Key laboratory of Polymorphic Drugs of Beijing, Institute of Materia Medica, Chinese Academy of Medical Sciences & Peking Union Medical College, Beijing, China

***Correspondence:**

Zhufang Shen, E-mail: shenzhf@imm.ac.cn

Caina Li, E-mail: [leecaina@imm.ac.cn](mailto:leecaina@imm.ac.cn)

^†^ Hui Cao and Caina Li contributed equally to this work.


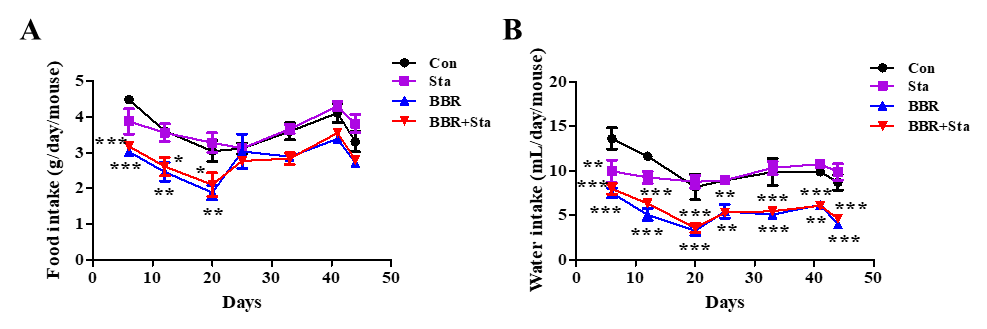


**Figure S1 Effects of berberine with stachyose on food intake and water intake.** **(A)**. Food consumption. **(B)**. Water consumption. Data are expressed as mean ± SEM (n=10-12). *p < 0.05, **p < 0.01, ***p < 0.001 vs. Con. Con, control; Sta, stachyose; BBR, berberine.


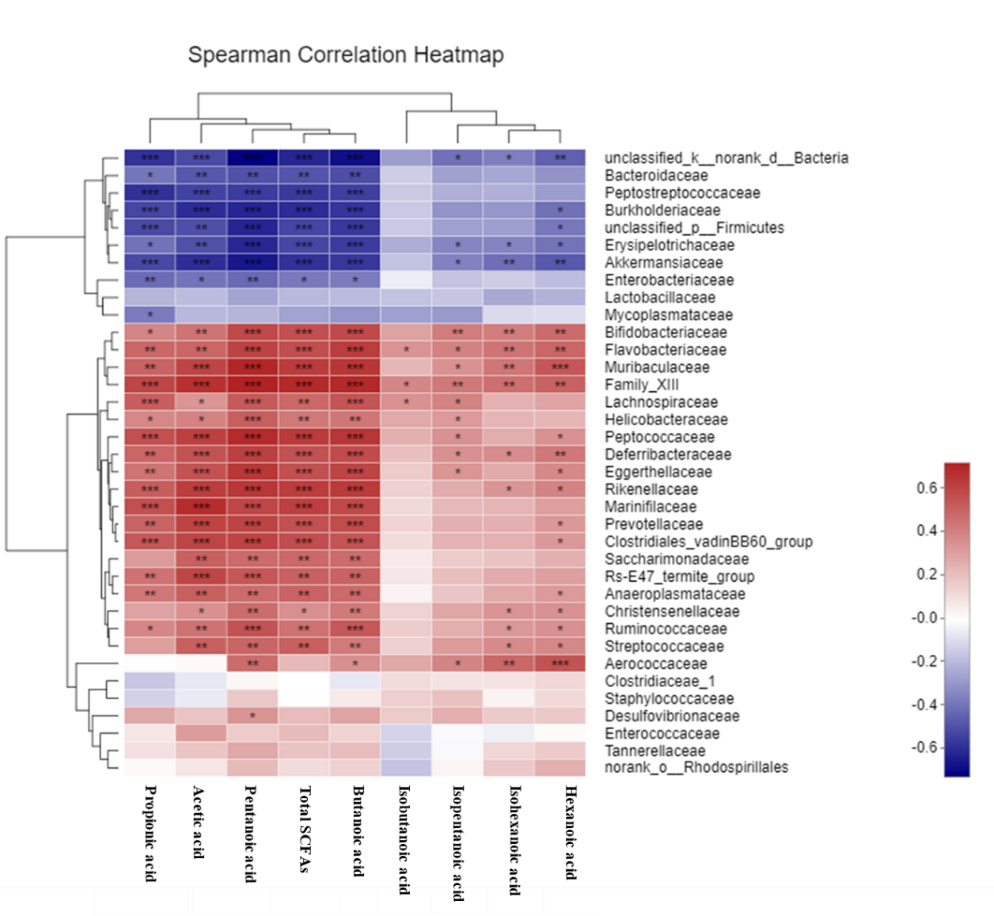


**Figure S2. Correlation analysis of SCFAs and gut microbiota at the family level.** R value was shown in different colors in the diagram. R and P values were obtained through Spearman’s rank correlation on the platform of Majorbio I-Sanger Cloud. The blue represents negative correlation, and red represents positive correlation. *p < 0.05, **p < 0.01, ***p < 0.001.

**Table S1 Primer sequences for qRT-PCR**

| **Gene** | **Primer sequence (5’—3’)** |
| --- | --- |
| IL-1β | Forward: CCAGCTTCAAATCTCACAGCAG  Reverse: CTTCTTTGGGTATTGCTTGGGATC |
| IL-6 | Forward: ACAACGATGATGCACTTGCAGA  Reverse: GATGAATTGGATGGTCTTGGTC |
| IL-10 | Forward: TGGCCCAGAAATCAAGGAGC  Reverse: CAGCAGACTCAATACACACT |
| MCP-1 | Forward: CCCTAGAAGCTCACCAAGGC  Reverse: GGTCTAGCCTCAACACCACC |
| Muc2 | Forward: CCTGAAGACTGTCGTGCTGT  Reverse: GGGTAGGGTCACCTCCATCT |
| Muc3 | Forward: GCTGGCTTTCATCCTCCACT  Reverse: CCTCCATCCCACACACTTCC |
| Occludin | Forward: ATGTCCGGCCGATGCTCTC  Reverse: TTTGGCTGCTCTTGGGTCTGTAT |
| Reg3g | Forward: TTCCTGTCCTCCATGATCAAAA  Reverse: CATCCACCTCTGTTGGGTTCA |
| TLR4 | Forward: CGCTTTCACCTCTGCCTTCACTACAG  Reverse: ACACTACCACAATAACCTTCCGGCTC |
| TNF-α | Forward: CACAGAAAGCATGATCCGCGACGT  Reverse: CGGCAGAGAGGAGGTTGACTTTCT |
| ZO-1 | Forward: ACCCGAAACTGATGCTGTGGATAG  Reverse: AAATGGCCGGGCAGAACTTGTGTA |
| β-actin | Forward: TGTGATGGTGGGAATGGGTCAG  Reverse: TTTGATGTCACGCACGATTTCC |
